# Hybrid-argon plasma coagulation for superficial esophageal cancer at risk of airway obstruction: a case report

**DOI:** 10.1055/a-2784-8170

**Published:** 2026-02-13

**Authors:** Junki Toyoda, Aiji Hattori, Yohei Ikenoyama, Misaki Nakamura, Yasuhiko Hamada, Noriyuki Horiki, Hayato Nakagawa

**Affiliations:** 1 220937 Department of Gastroenterology and Hepatology, Mie University Hospital, Tsu, Japan

Endoscopic submucosal dissection (ESD) is the standard treatment for superficial esophageal squamous cell carcinoma (ESCC). However, this procedure is associated with complications and a relatively long procedure time 1 . Argon plasma coagulation (APC) is a technically simple treatment for ESCC, but APC can sometimes cause esophageal perforation or stricture 2 3 . Hybrid APC (H-APC), which combines APC with high-pressure submucosal injection, has recently been developed. This technique enables controlled coagulation with improved safety and precision ( 4 5 Fig. 1 ).

**Fig. 1:**
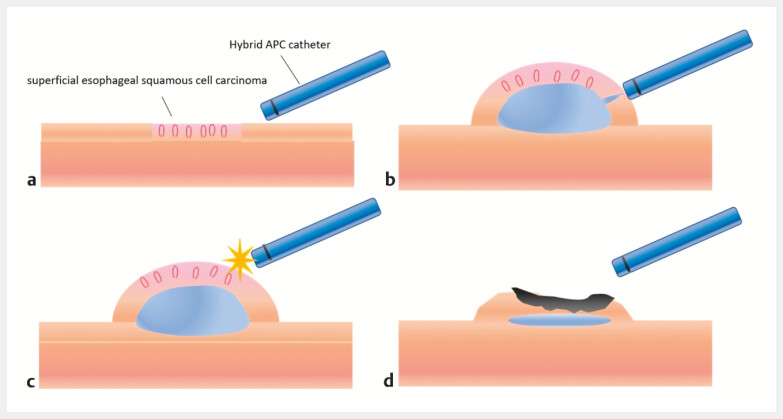
**a** The APC catheter was positioned in contact with the mucosal surface. **b** A submucosal fluid cushion was created using a high-pressure, needleless injection. **c** Following the injection, thermal ablation was performed on the ESCC. **d** The presence of a submucosal cushion may help enhance the safety of the thermal ablation procedure. Adapted from Hattori A, Ikenoyama Y, Fujiwara Y, et al. Endoscopy 2025; 57: E987–E988. APC, argon plasma coagulation; ESCC, esophageal squamous cell carcinoma.

A 59-year-old male patient was diagnosed with superficial ESCC with significant laryngeal edema due to a prior history of radiation therapy for laryngeal cancer. Due to the high risk of airway obstruction associated with prolonged sedation, H-APC was selected as a simpler and faster treatment for ESCC ( Fig. 2 ).

**Fig. 2:**
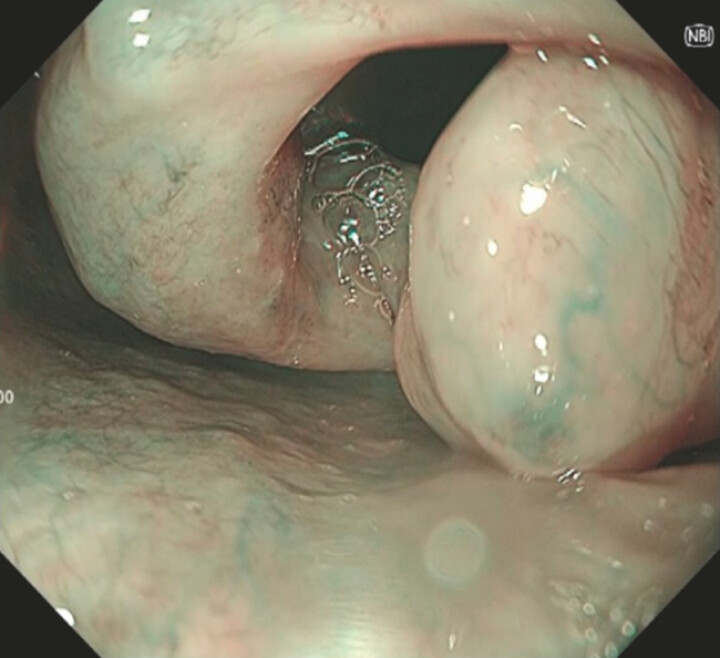
Significant laryngeal edema was observed during endoscopic evaluation.

A thin-diameter endoscope (EG-840TP; Fujifilm, Tokyo, Japan) was used because of pharyngeal stenosis caused by prior radiation. Following lesion marking, a submucosal cushion was created using H-APC by injecting a saline solution mixed with indigo carmine. Thermal coagulation was performed using the pulsed APC slow 5.0 (ERBE JET2, effect 40). Initial coagulation with H-APC facilitated the detachment of the superficial squamous epithelium. The loosened epithelial layer was then easily removed using an endoscope. A second submucosal injection was subsequently administered using H-APC, and coagulation was reapplied to enhance submucosal ablation. The procedure time from the initial submucosal injection to the completion of coagulation was 8 minutes ( Fig. 3 , Video 1 ). No immediate complications, such as perforations, were observed. Follow-up endoscopy at 2 months showed complete scar formation with no endoscopic evidence of residual or recurrent lesions.

**Fig. 3:**
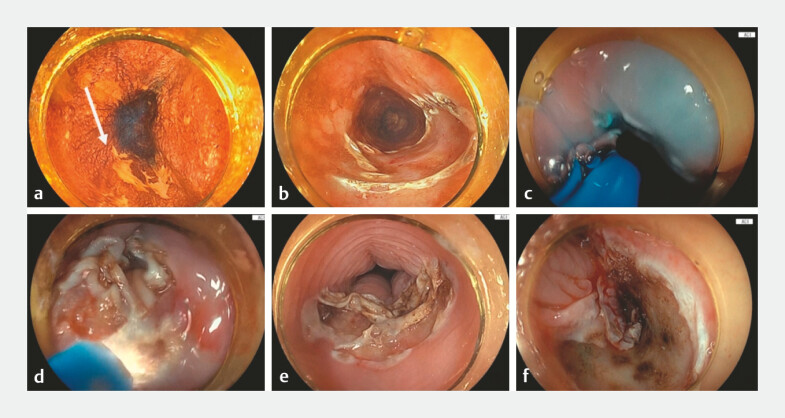
**a** ESCC was detected in the mid-thoracic esophagus. **b** The lesion margins were initially marked using H-APC. **c** Following lesion marking, a submucosal cushion was created by injecting a saline solution mixed with indigo carmine using H-APC. **d** ESCC was being cauterised using H-APC. **e** Initial coagulation with H-APC facilitated the detachment of the superficial squamous epithelium. The loosened epithelial layer was then easily removed using the endoscope. **f** A second submucosal injection was subsequently administered using H-APC, and coagulation was reapplied to enhance submucosal ablation. ESCC, esophageal squamous cell carcinoma; H-APC, hybrid argon plasma coagulation.

Hybrid argon plasma coagulation (H-APC) procedure for superficial esophageal squamous cell carcinoma.Video 1

H-APC was performed safely and efficiently. H-APC could potentially serve as an effective therapeutic option for ESCC cases that are challenging to manage with ESD.

Endoscopy_UCTN_Code_TTT_1AO_2AN

## References

[1] Guo HM Zhang XQ Chen M Endoscopic submucosal dissection vs endoscopic mucosal resection for superficial esophageal cancer World J Gastroenterol 2014 20 5540 5547 10.3748/wjg.v20.i18.5540 24833885 PMC4017070

[2] TaharaKTanabe S Ishido K Argon plasma coagulation for superficial esophageal squamous-cell carcinoma in high-risk patients World J Gastroenterol 2012 18 5412 5417 10.3748/wjg.v18.i38.5412 23082058 PMC3471110

[3] Chung CH Chou TC Chen CY Minute perforation after argon plasma coagulation for management of small colonic polyps Endoscopy 2009 41 E251 E252 19787576 10.1055/s-0029-1214432

[4] Davide M Roberta M Silvia P Efficacy and safety of H-APC in Barrett's esophagus: Italian prospective multicenter study Endosc Int Open 2025 13 a25318227 40018075 10.1055/a-2531-8227 PMC11866041

[5] Hattori A Ikenoyama Y Fujiwara Y Hybrid argon plasma coagulation for chronic radiation-induced proctitis following pelvic chemoradiotherapy for cervical adenocarcinoma: a case report Endoscopy 2025 57 E987 E988 10.1055/a-2686-7678 40907550 PMC12411006

